# Supplementary material for: Single cell transcriptional and chromatin accessibility profiling redefine cellular heterogeneity in the adult human kidney
Source: Nat Commun. 2021 Apr 13;12:2190. doi: 10.1038/s41467-021-22368-w (PMC8044133; doi:10.1038/s41467-021-22368-w)
Supplement: Supplementary file 1 — Supplementary Information [file 41467_2021_22368_MOESM1_ESM.pdf]

Supplementary Information

Single cell transcriptional and chromatin accessibility profiling redefine cellular heterogeneity in the adult human kidney

Yoshiharu Muto, Parker C. Wilson, Nicolas Ledru, Haojia Wu, Henrik Dimke, Sushrut S. Waikar and Benjamin D. Humphreys

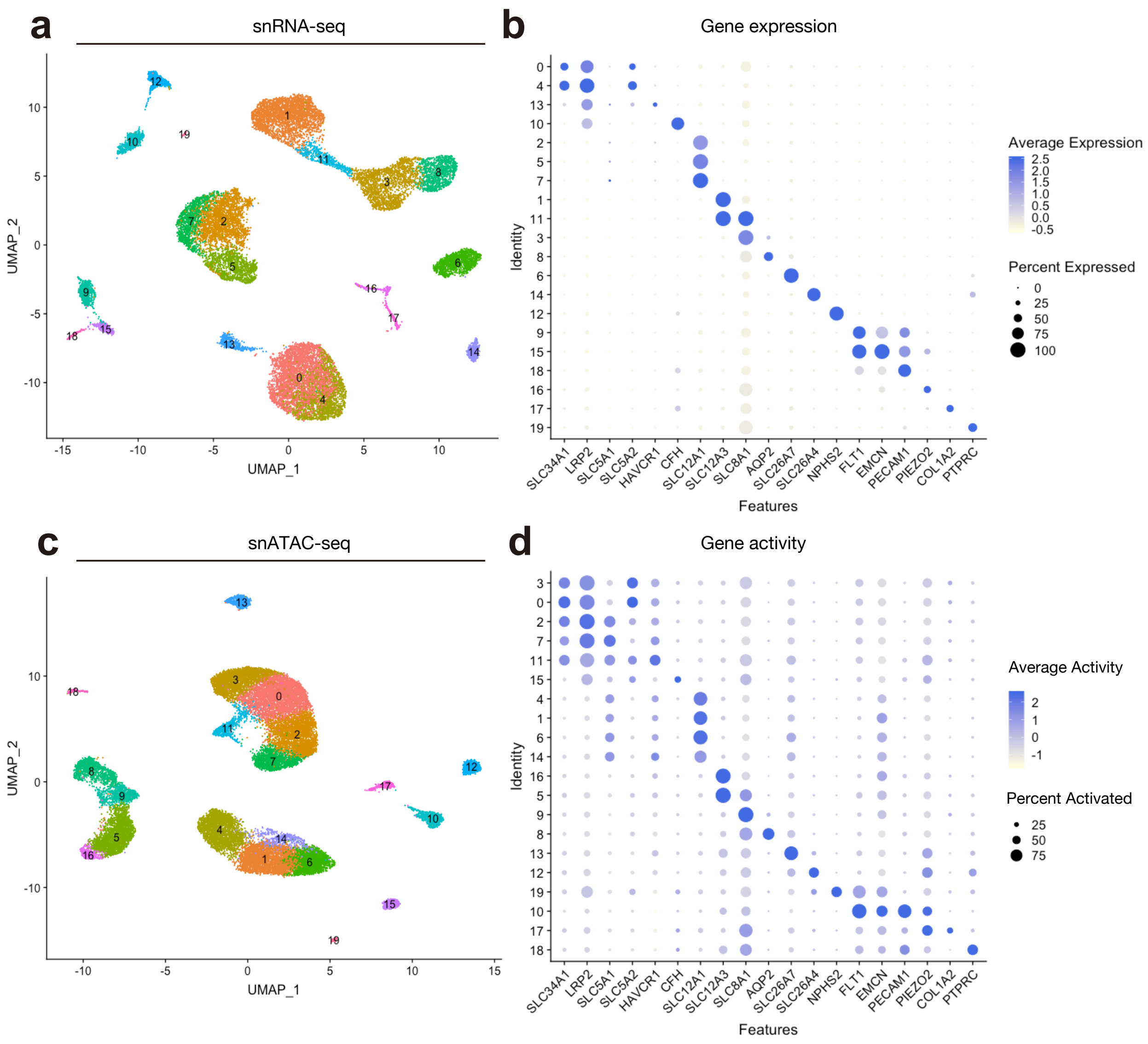

Supplementary Figure 1 – Unsupervised clustering of snRNA-seq and snATAC-seq human kidney datasets: (**a**) Unsupervised clustering of snRNA-seq dataset in Seurat, and (**b**) the dot plots showing marker gene expressions of each cell types. The diameter of the dot corresponds to the proportion of cells expressing the indicated gene and the density of the dot corresponds to average expression relative to all cell types. (**c**) Unsupervised clustering of snATAC-seq dataset in Signac, and (**d**) the dot plots showing marker gene activities of each cell types. The diameter of the dot corresponds to the proportion of cells with detected activity of indicated gene and the density of the dot corresponds to average gene activity relative to all cell types.

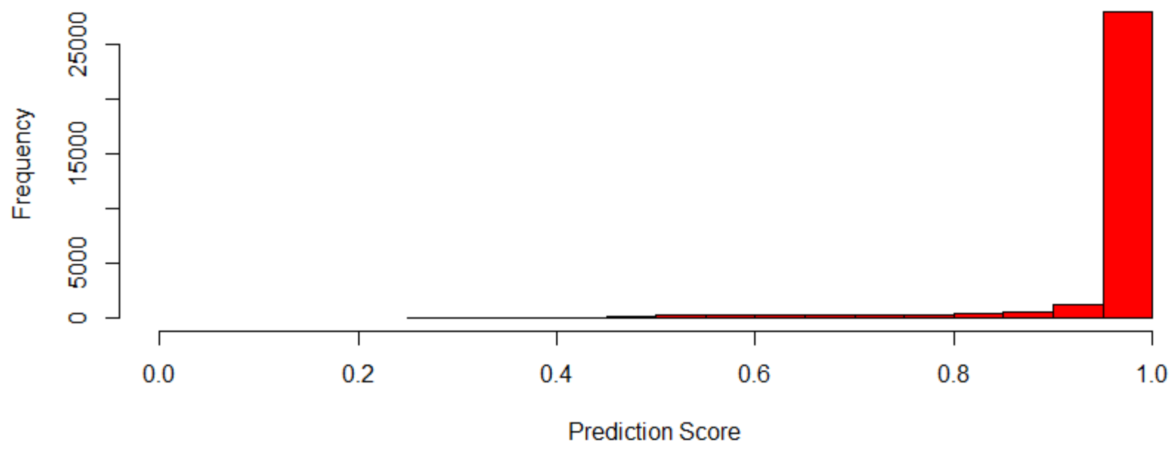

Supplementary Figure 2 – Label transfer of annotated snRNA-seq confidently predicts snATAC-seq cell types: Distribution of maximum prediction scores of nuclei calculated by the label transfer algorithm in Signac package. A gene activity matrix was created from the snATAC-seq data and transfer anchors were identified between the 'reference' snRNA-seq dataset and 'query' gene activity matrix followed by assignment of predicted cell types using the Signac package.

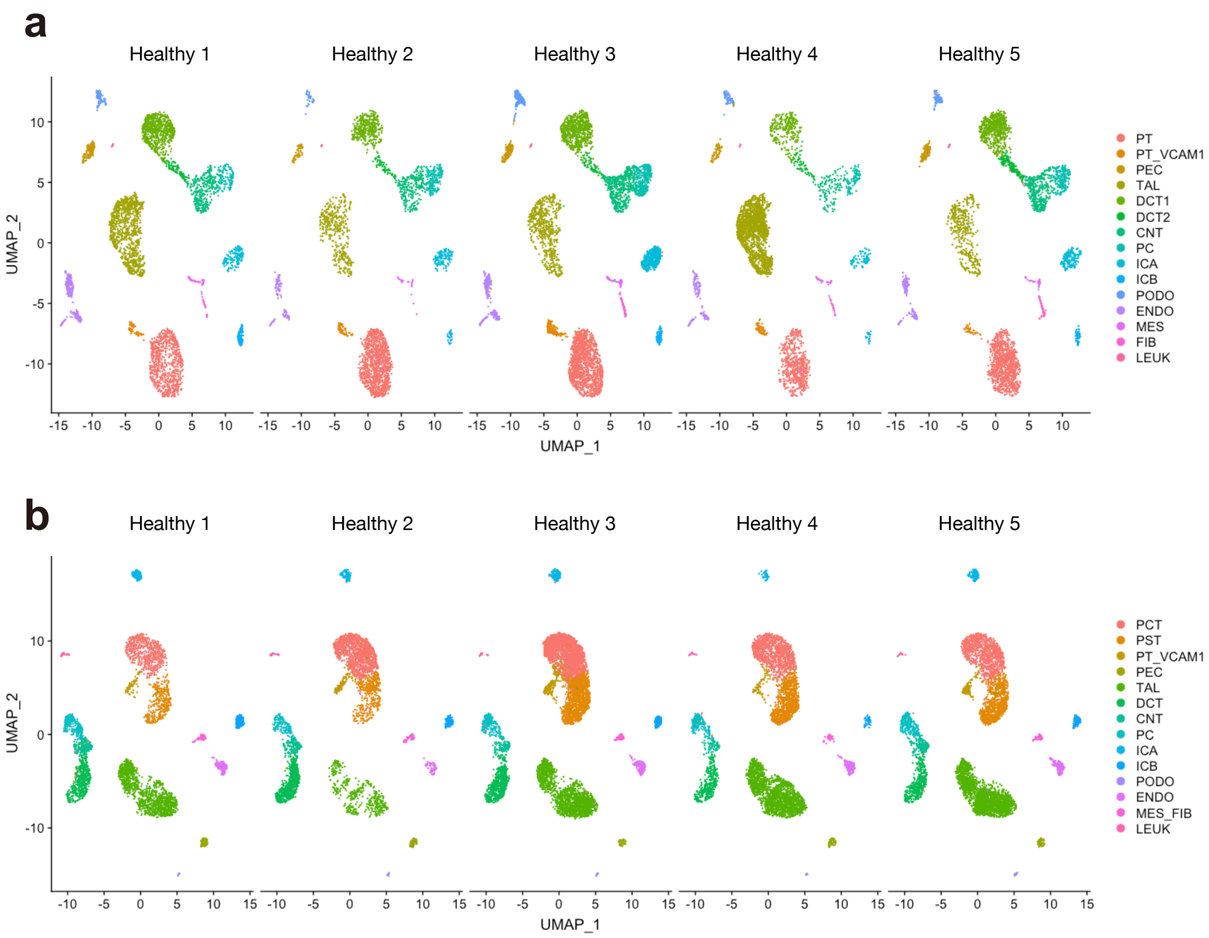

Supplementary Figure 3 – All cell types detected are found in each kidney sample in both modalities: (**a**) UMAP visualization of snRNA-seq dataset per kidney sample. (**b**) UMAP visualization of snATAC-seq dataset per kidney sample.

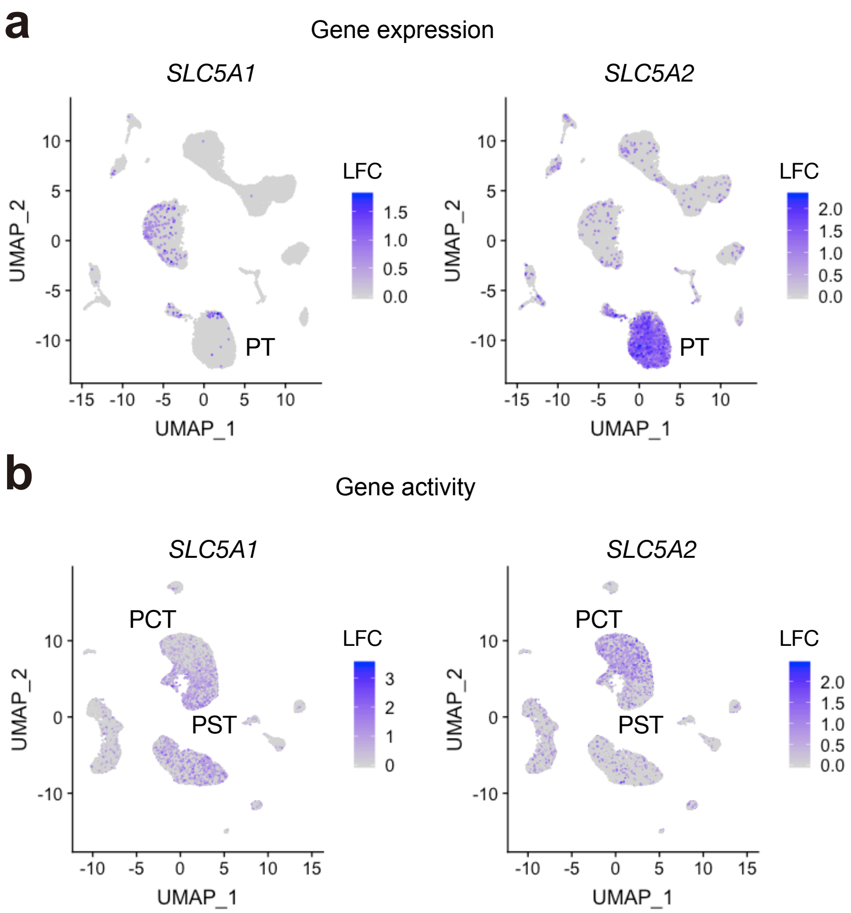

Supplementary Figure 4 – *SLC5A1* and *SLC5A2* gene activity delineate proximal tubule segments: (**a**) snRNA-seq does not detect *SLC5A1* expression, and *SLC5A2* expression does not clearly distinguish subpopulations of proximal tubule. (**b**) snATAC-seq shows increased *SLC5A1* gene activity in a subpopulation of proximal tubule (PST) that is mutually exclusive to the subpopulation (PCT) showing increased *SLC5A2* gene activity. The color scale for each plot represents a normalized log-fold-change (LFC).

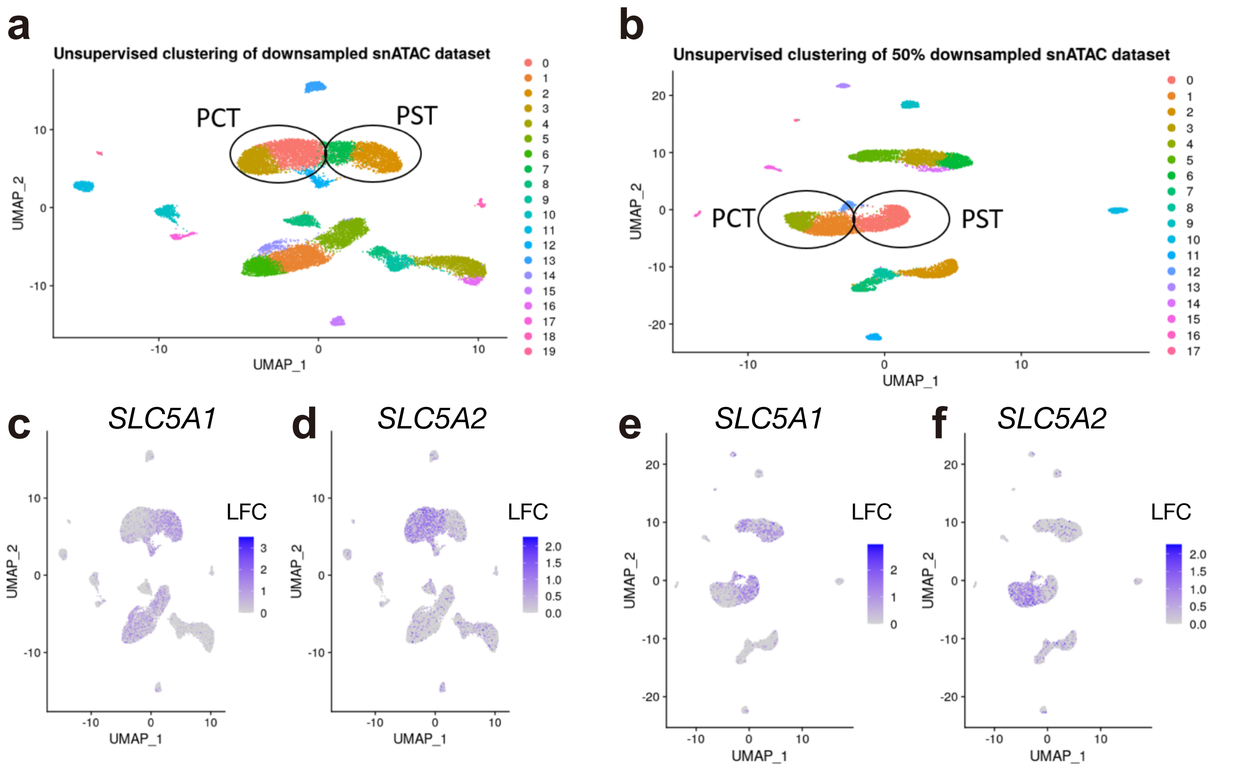

Supplementary Figure 5 – *SLC5A1* and *SLC5A2* gene activity delineate proximal tubule segments after downsampling: Resolving the difference between the proximal convoluted tubule (PCT, high *SLC5A2* expression) and proximal straight tubule (PST, high *SLC5A1* expression) subclusters in the snATAC-seq dataset after downsampling to the same number of cells as the snRNA dataset (19,985 cells, **a**) and 50% of the cells in the snRNA dataset (9,992 cells, **b**). A umap plot displaying *SLC5A1* or *SLC5A2* gene activity in each downsampled dataset are also shown (**c-f**). The color scale for each plot represents a normalized log-fold-change (LFC).

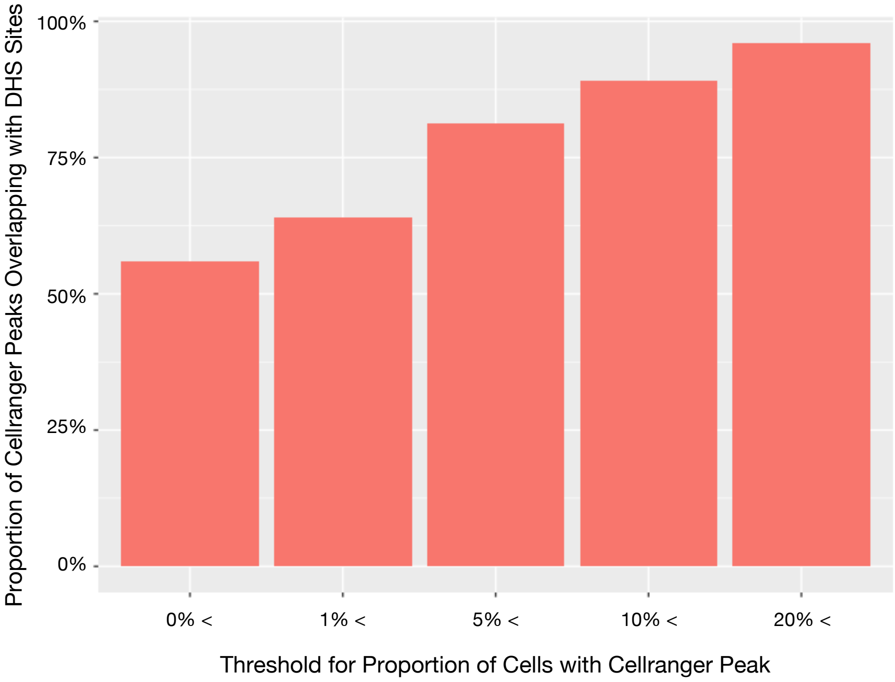

Supplementary Figure 6 – Aggregated Cell Ranger peaks significantly overlap with previously-published DNase hypersensitive sites: Cell Ranger peaks were filtered for peaks contained in a designated proportion of cells (x-axis). DNase hypersensitive sites (DHS) were downloaded from Sieber et al. (PMID: 30760496) and overlapped with the Cell Ranger peaks using the GenomicRanges package. The proportion of overlap between DHS and Cell Ranger peaks increased as Cell Ranger peaks were filtered for peaks present in an increasing proportion of cells.

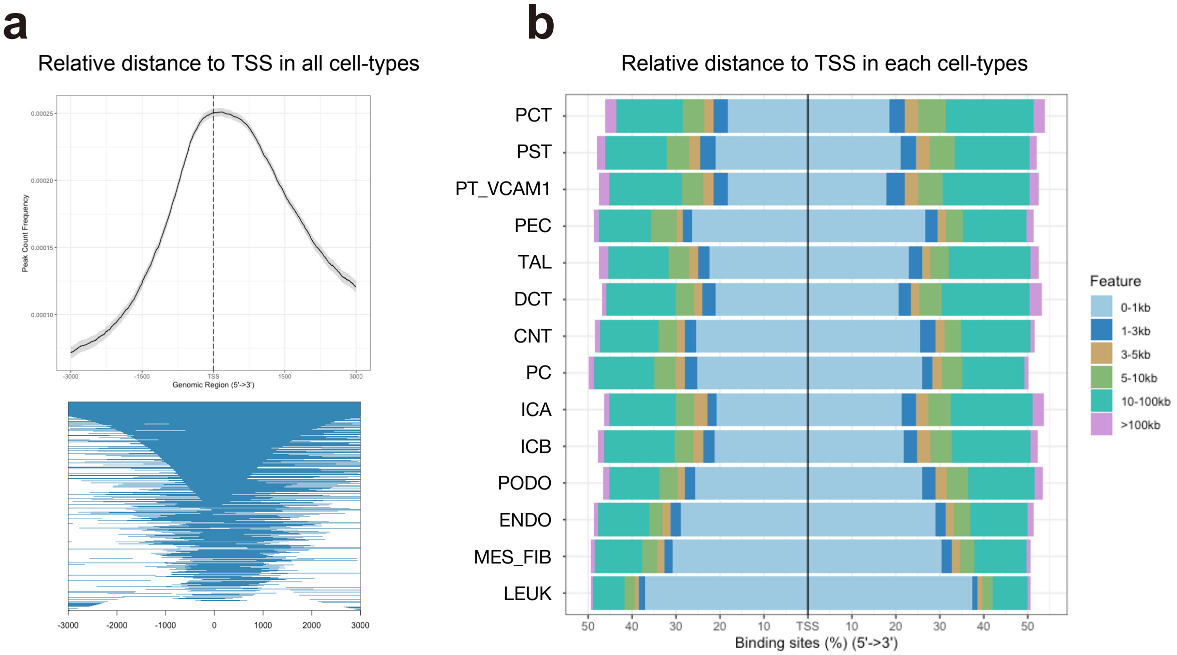

Supplementary Figure 7 – Cell type-specific DAR are enriched around the transcription start sites (TSS): (**a**) Relative distance of differentially accessible region (DAR) to TSS in all the dataset. (**b**) Relative distance of DAR to TSS in each cell type.

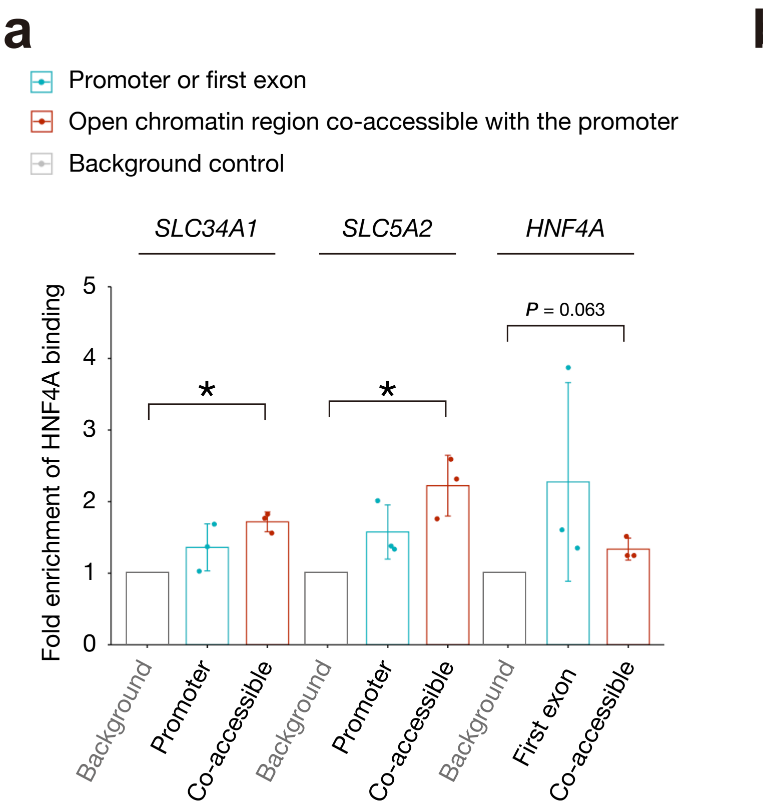

Target	Coordinates
Background control	chr5:177393216-177393337
SLC34A1 promoter	chr5:177384377-177384389
SLC34A1 co-accessible	chr5:177379077-177379089
SLC5A2 promoter	chr16:31482945-31482957
SLC5A2 co-accessible	chr16:31458141-31458153
HNF4A first exon	chr20:44401447-44401459
HNF4A co-accessible	chr20:44395660-44395672

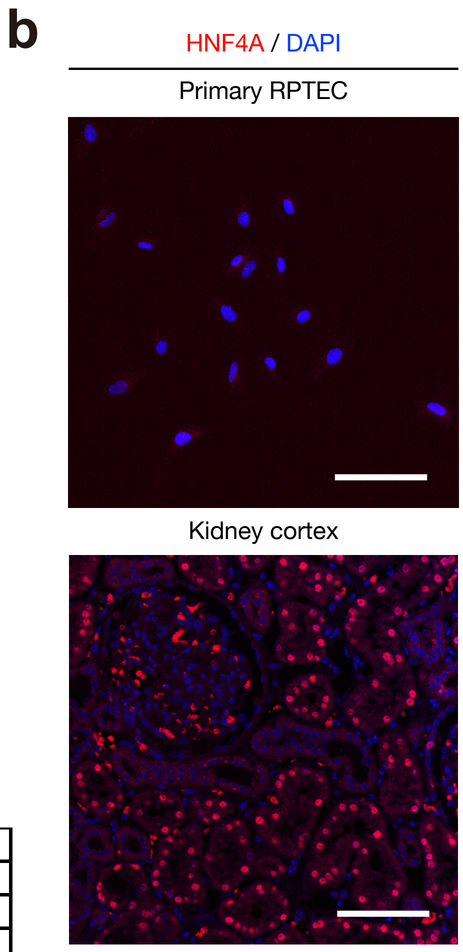

Supplementary Figure 8 – HNF4A binds the predicted HNF4A motifs within DAR for selected target gene loci in RPTEC: (**a**) ChIP followed by quantitative PCR (ChIP-qPCR) analysis of HNF4A binding within the promoter, first exon or the open chromatin regions that were predicted to interact with promoters via a CCAN in the differentially expressed gene loci (*SLC34A1*, *SLC5A2* and *HNF4A*) in RPTEC (n = 3 independent samples). ChIP-qPCR was performed with an open chromatin region without HNF4A motif on the intronic region of *SLC34A1* gene as a background control. Data are mean±s.d. *P<0.05 (P = 0.0122, 0.0378, two-sided one sample t-test). The table shows the ChIP-qPCR target regions and their coordinates. (**b**) Representative immunostaining images of HNF4A (red) in the kidney cortex or primary RPTEC in the primary RPTEC. Scale bar indicates 100 µm. Two independent experiments were performed, and similar results were obtained.

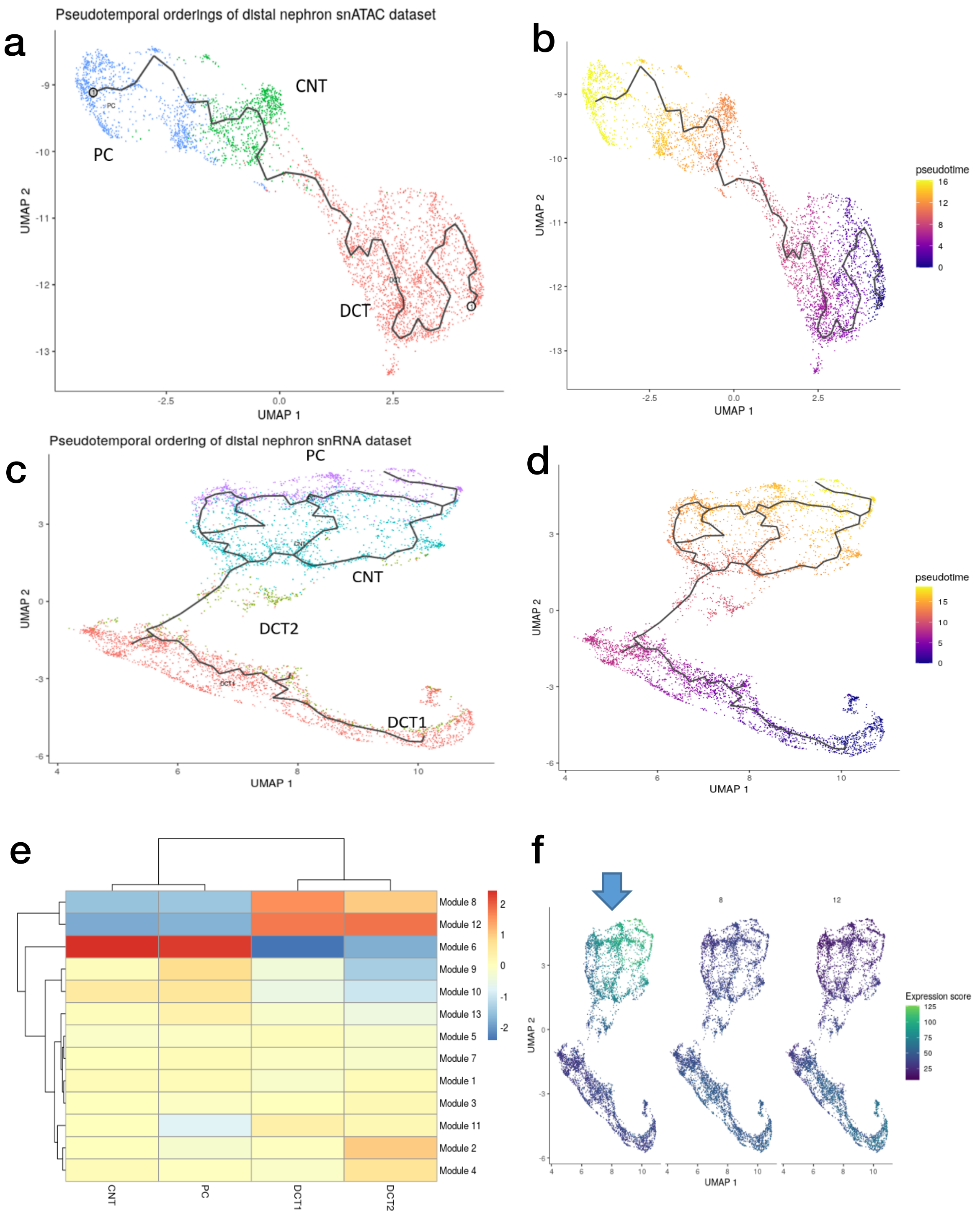

Supplementary Figure 9 - Pseudotime-dependent gene modules that are significantly up- or down-regulated in the distal nephron progressing from proximal to distal. (**a,b**) Pseudotemporal ordering of distal nephron cells in snATAC-seq data. (**c,d**) Pseudotemporal ordering of distal nephron cells in snRNA-seq data. (**e**) Gene modules associated with pseudotemporal ordering of the snRNA dataset. (**f**) Module 6,8 and 12 were visualized where they lay along the pseudotemporal trajectory. Module 6 showed the highest activity in PC and CNT among all gene modulles.

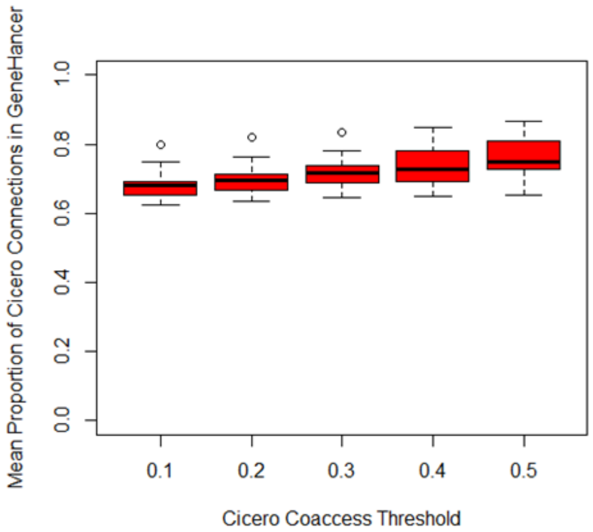

Supplementary Figure 10 – Cicero connections significantly overlap with the GeneHancer interaction database: The snATAC-seq dataset was partitioned into individual cell types and cell-type-specific cis-coaccessibility networks (CCAN) were identified with the R package Cicero. Cicero connections within 50kb of a cell-type-specific differentially accessible region (DAR) were compared to GeneHancer 'double elite' interactions downloaded from the UCSC table browser for varying Cicero coaccessibility thresholds, and the percentage of overlapped interactions are shown. Box-and-whisker plots depict the median, quartiles and range.

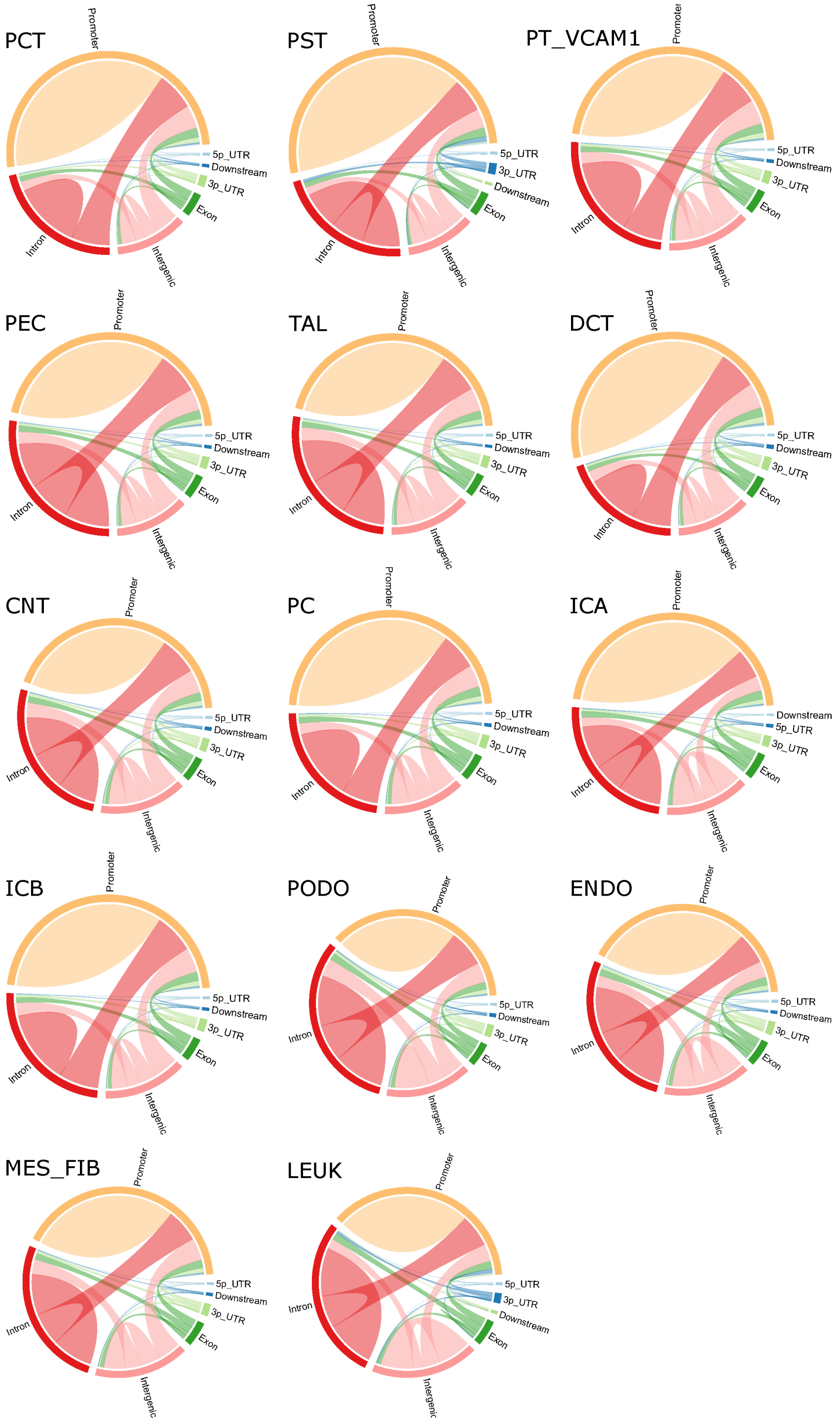

Supplementary Figure 11 – Annotation of Cicero Connections: The snATAC-seq dataset was partitioned into individual cell types and cis-coaccessibility networks were predicted with Cicero. The Cicero connection endpoints with a coaccessibility threshold > 0.2 were annotated with ChIPSeeker using the UCSC database. The relative number of connections within and between the designated genomic regions is displayed for each cell type. Promoter - region within 3kb of the transcriptional start site. 3p_UTR- 3’ untranslated region, 5p_UTR- 5’ untranslated region, Downstream - 3kb downstream of the 3’ UTR.

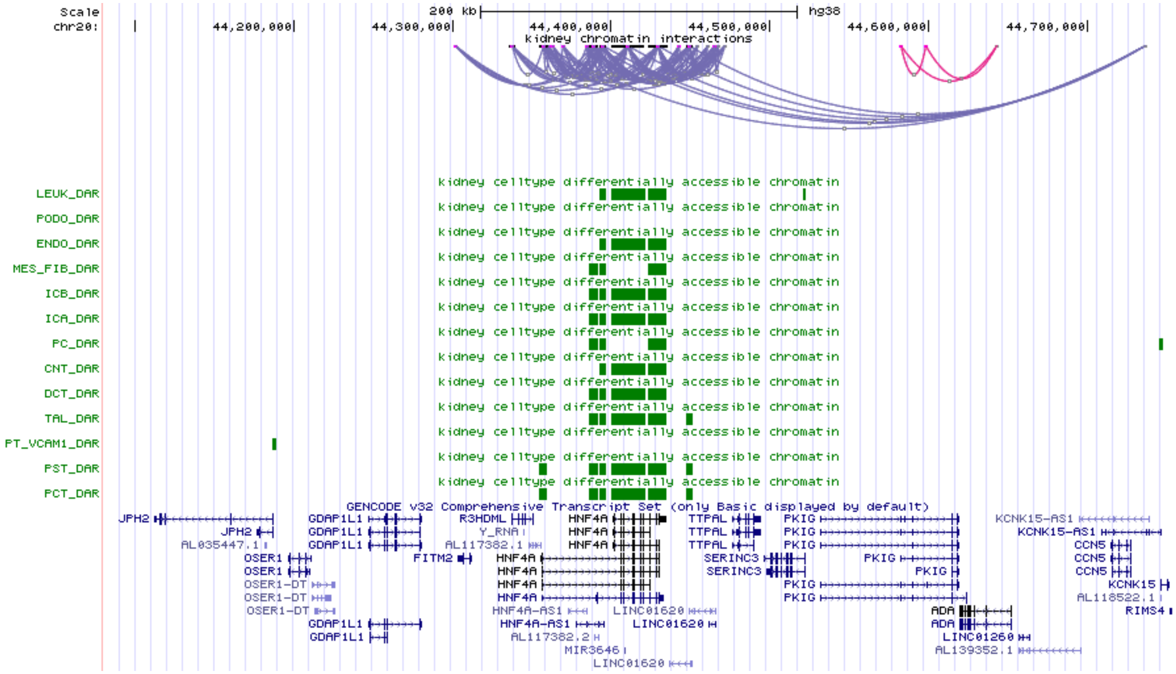

Supplementary Figure 12 – Visualization of cell-type-specific differentially accessible chromatin and cis-coaccessibility networks with the UCSC genome browser: Accessible chromatin regions for each cell type (labels along left hand column) are indicated by green boxes. Blue and pink arcs along the top of the image indicate consensus predicted chromatin interactions near the *HNF4A* locus and are colored by cis-coaccessibility network. The depicted tracks can be loaded by clicking on this link: https://genome.ucsc.edu/s/parkercwilson/control_celltype_cr . Alternatively, UCSC tracks are available in the Supplementary data for all cell types and can be uploaded to the genome browser using the My Data > Custom Tracks tab at http://genome.ucsc.edu.

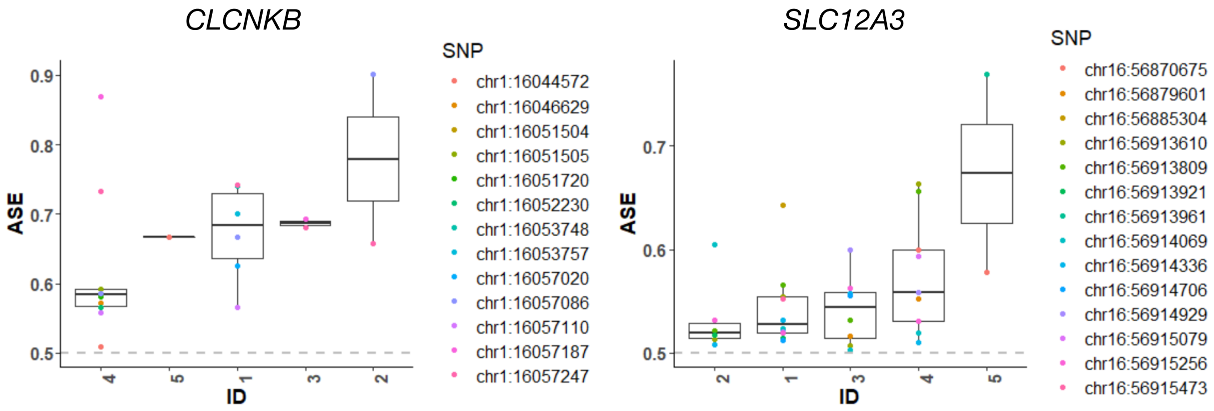

Supplementary Figure 13 - Allele-specific expression of *CLCNKB* and *SLC12A3* among all cell types in the snRNA dataset: Estimated SNV-level allele-specific expression (ASE) for each variant and donor in *CLCNKB* (left) or *SLC12A3* (right) gene. ASE level was estimated as major allelic fraction after haplotype pseudo-phasing. Box-and-whisker plots depict the median, quartiles and range.

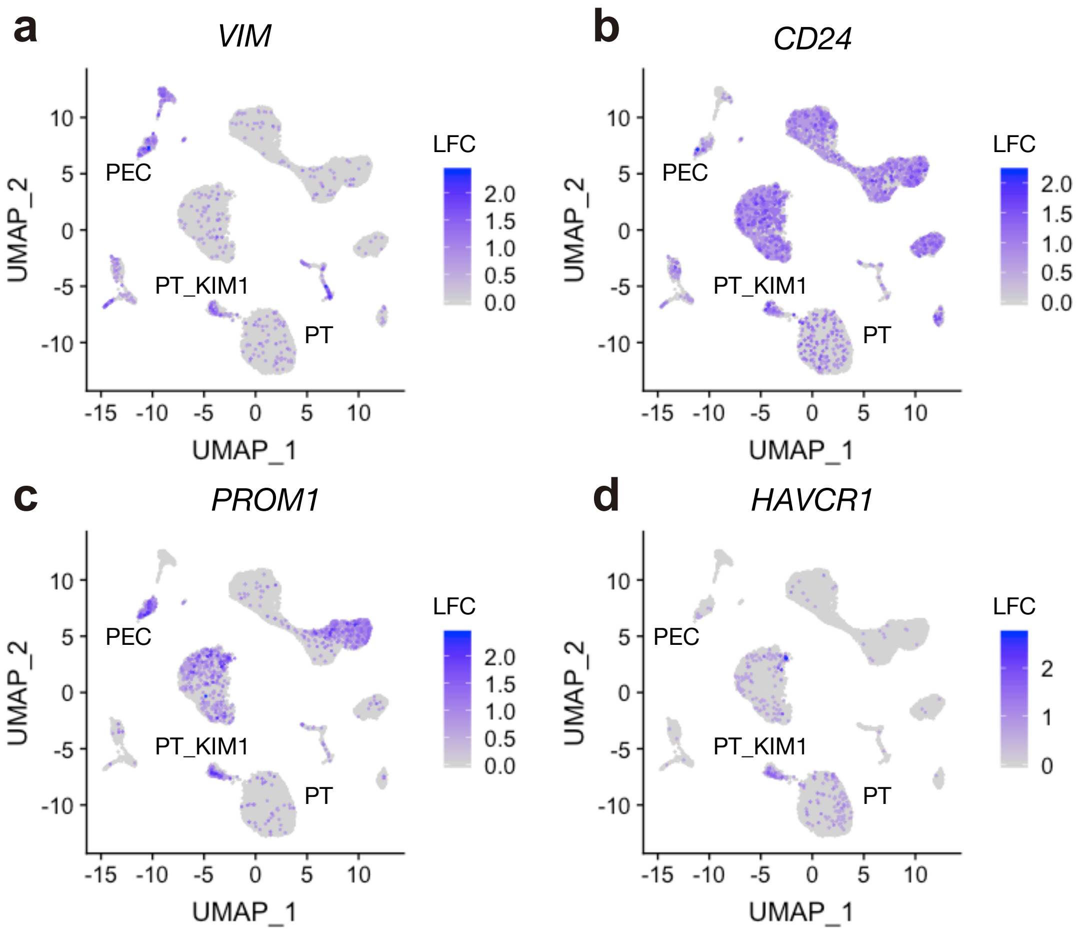

Supplementary Figure 14 – Marker gene expressions in the PT_VCAM1 population: (**a**) *VIM* (Vimentin) (**b**) *CD24*, (**c**) *PROM1* (CD133) and (**d**) *HAVCR1* expression in the snRNA-seq dataset shows increased expression of these genes in the PT_VCAM1 population compared to PT. The color scale for each plot represents a normalized log-fold-change (LFC).

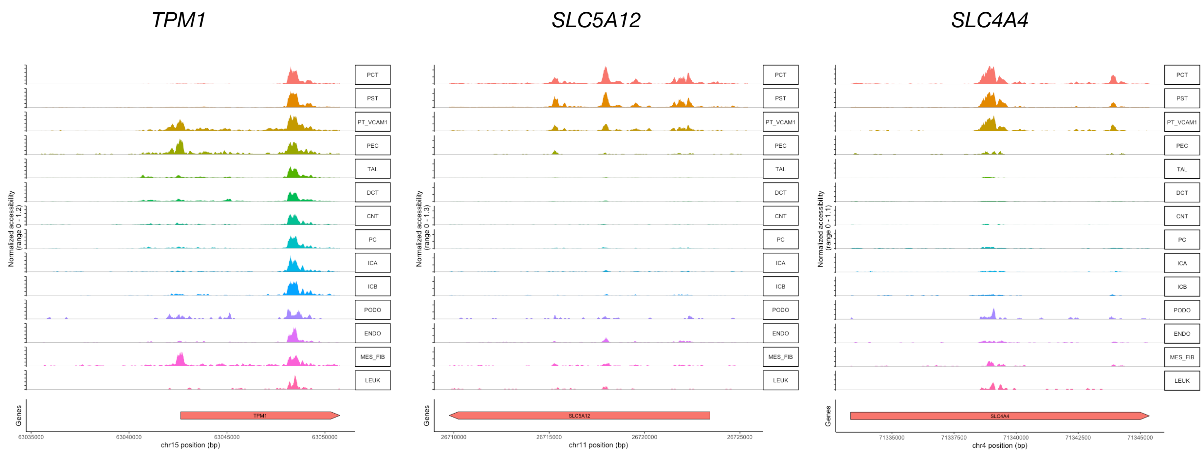

Supplementary Figure 15 – Fragment coverage around representative DAR of PT_VCAM1: Fragment coverage (frequency of Tn5 insertion) around the representative DAR (DAR +/-5000 bp) on *TPM1*, *SLC5A12* or *SLC4A4* locus shown in Fig.6C.

a

Hallmark gene set enriched in PT_VCAM1	NES	***P*-val**	FDR-***q***
TNFA_SIGNALING_VIA_NFKB	2.01	0.000	0.011
INTERFERON_GAMMA_RESPONSE	1.84	0.002	0.070
HYPOXIA	1.79	0.003	0.112
UV_RESPONSE_DN	1.77	0.002	0.127
EPITHELIAL_MESENCHYMAL_TRANSITION	1.76	0.003	0.148

b

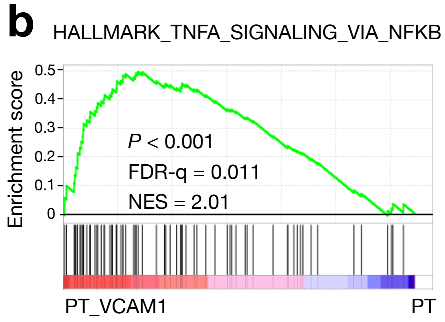

Supplementary Figure 16 – Gene set enrichment analysis on the differential expressed genes in PT_VCAM1 vs PT suggested activation of NF-κB pathway genes: GSEA of differentially expressed genes for hallmark gene sets (**a**) and the HALLMARK_TNFA_SIGNALING_VIA_NFKB gene set (genes regulated by NFκB induced by TNFα) (**b**) in PT_VCAM1 compared with PT. FDR false-discovery rate *q* value. NES normalized enrichment score. The pre-ranked gene list was analyzed and statistics were determined with GSEA v4.0.3 (Broad Institute).

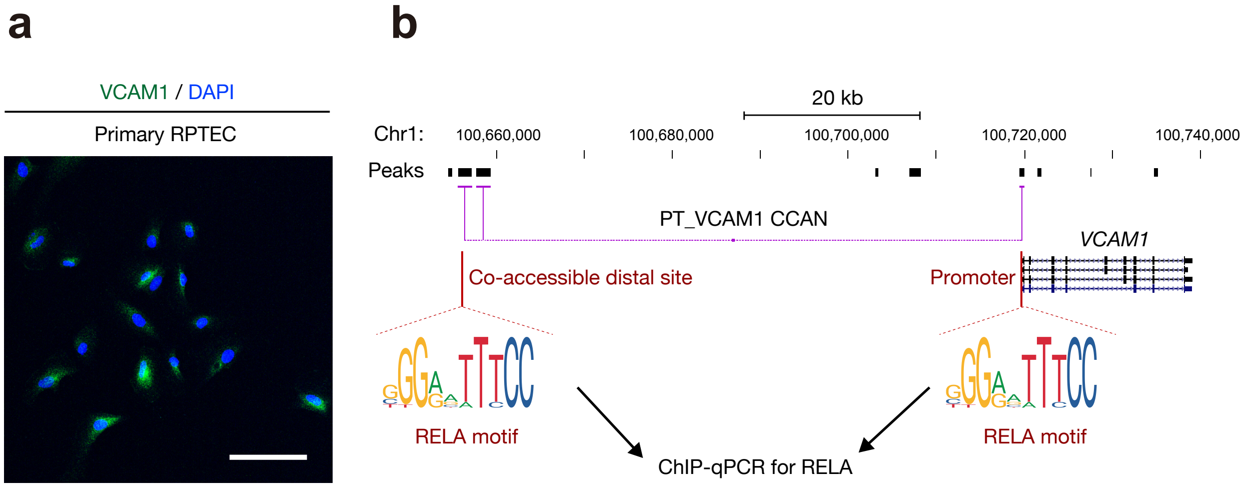

Supplementary Figure 17 – ChIP-qPCR for RELA was performed for the DAR on *VCAM1* locus in RPTEC: (**a**) Representative immunostaining images of VCAM1 (green) in the RPTEC. Scale bar indicates 100 µm. Two independent experiments were performed, and similar results were obtained. (**b**) Graphical experimental methodology for ChIP-qPCR analysis of RELA binding within the promoter or an open chromatin region that was predicted to interact with *VCAM1* promoter via a CCAN.

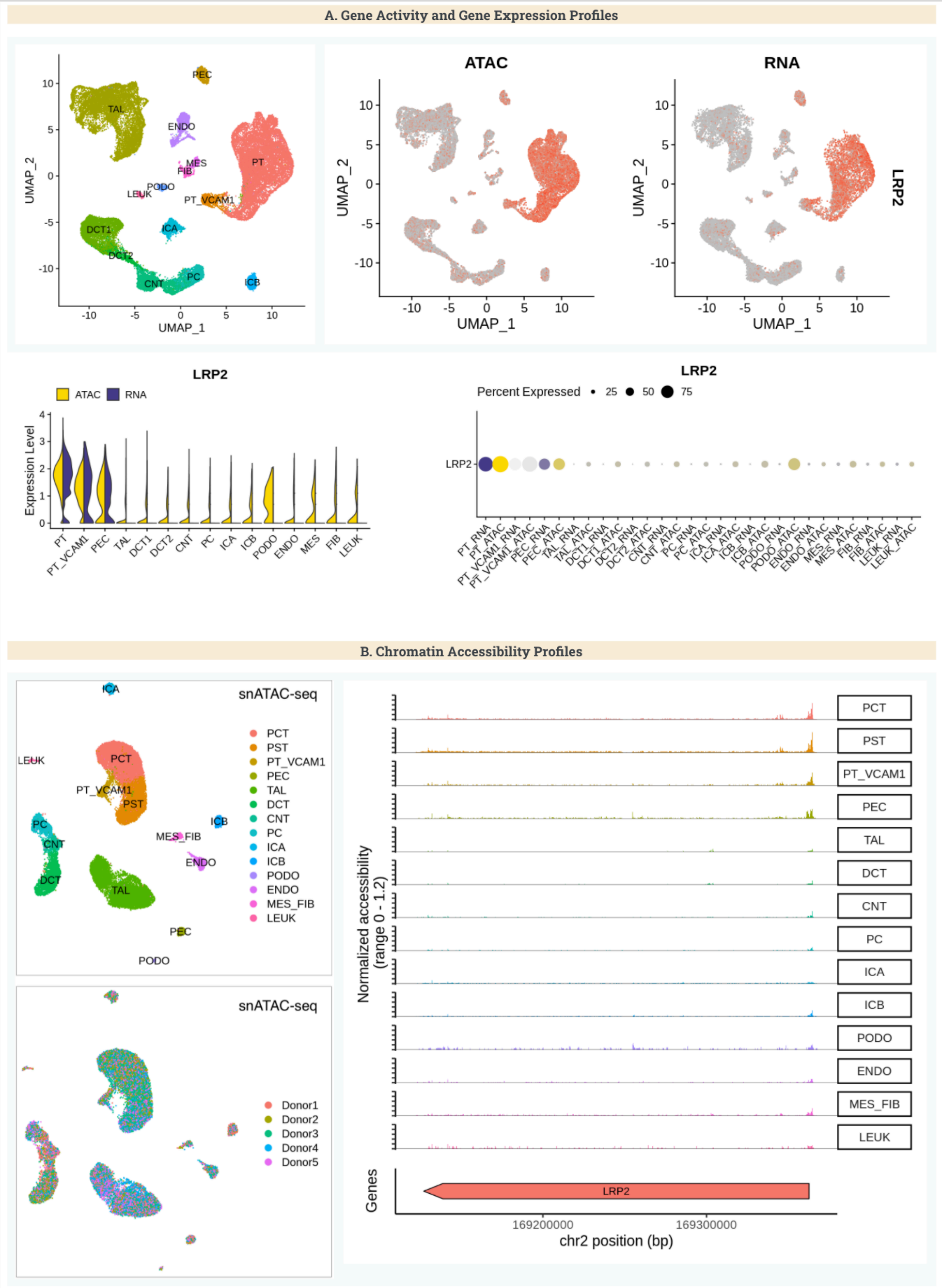

Supplementary Figure 18 – Online analyzer for the harmonized multimodal kidney cell atlas encompassing both transcriptomic and epigenomic data: Cell type-specific differential expressed genes, chromatin accessibilities, gene activities and predicted transcription factor motif activities are searchable on the webpage (http://humphreyslab.com/SingleCell/).

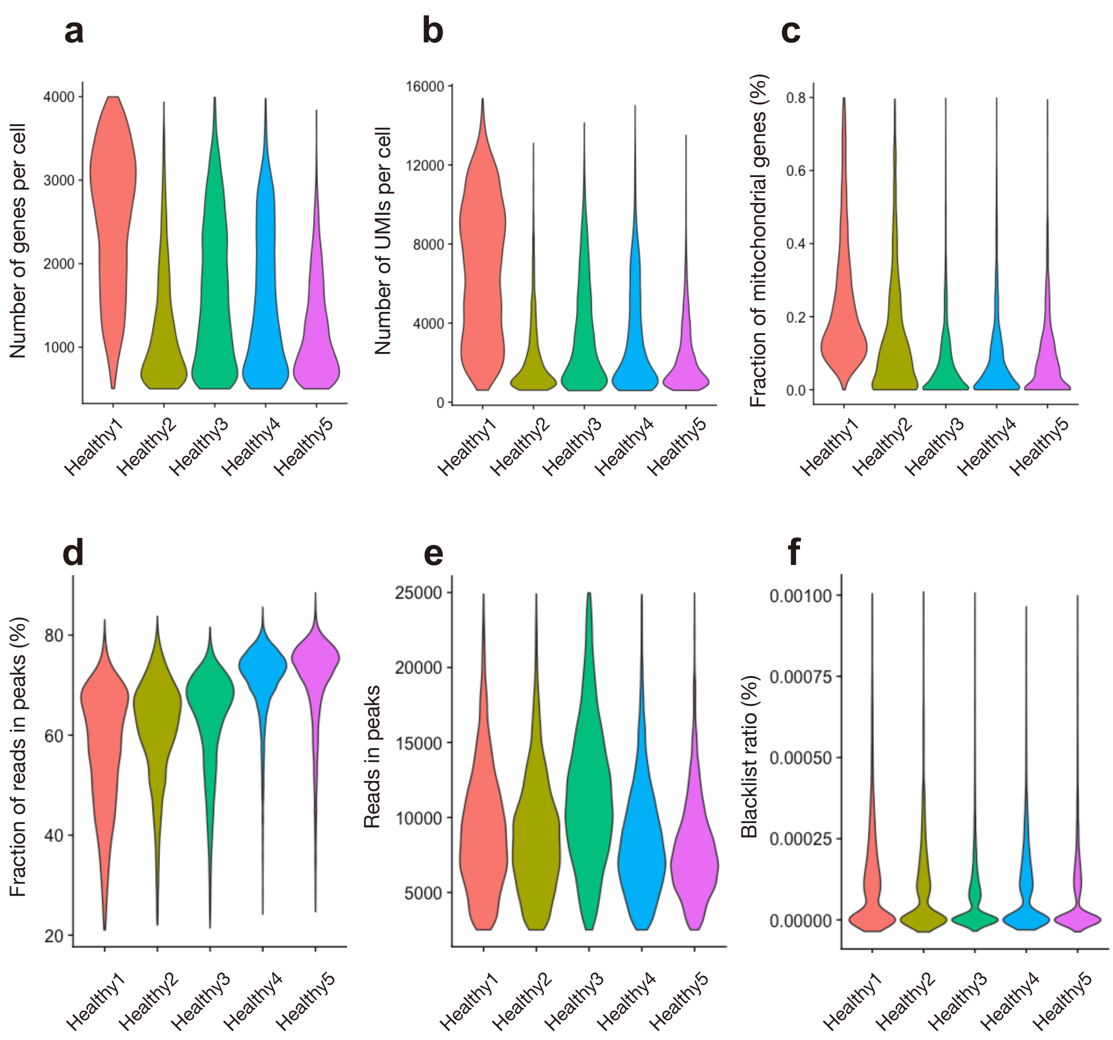

Supplementary Figure 19 – QC metrics for snRNA-seq or snATAC-seq dataset: (**a**) Number of genes per cell, (**b**) number of UMIs per cell and (**c**) fraction of mitochondrial genes per cell in snRNA-seq data were shown. (**d**) Fraction of reads in peaks, (**e**) number of reads in peaks per cell and (**f**) ratio of reads in genomic blacklist region per cell in snATAC-seq data were shown.

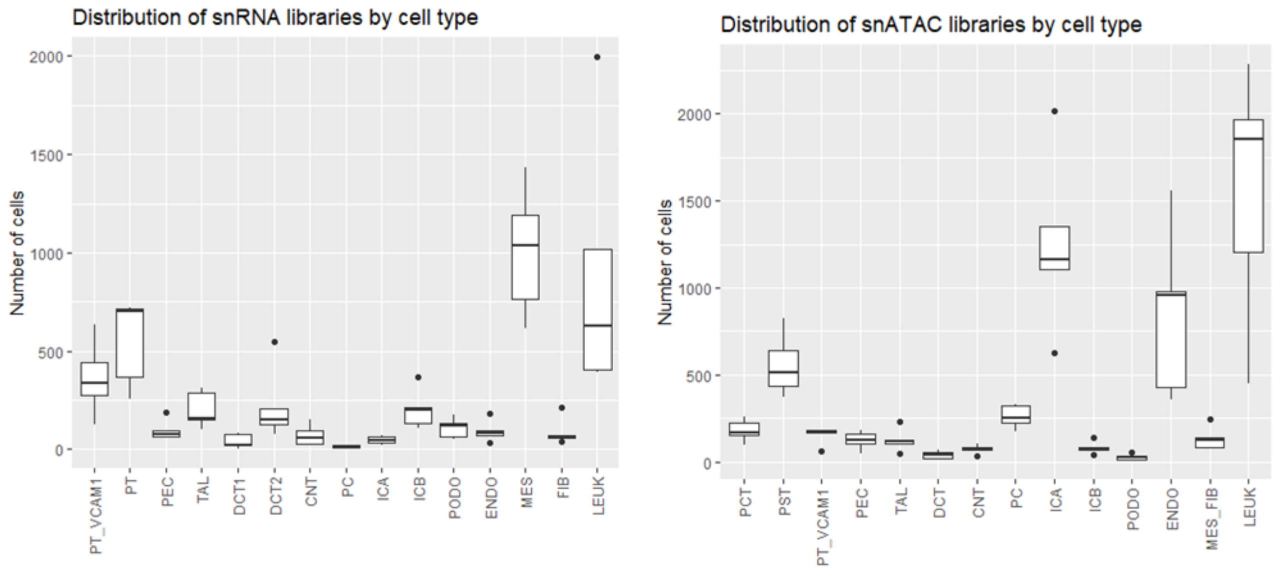

Supplementary Figure 20 – The number or frequency of cells for each cell type quantitated in the filtered snRNA-seq or snATAC-seq dataset – The number or frequency of cells for each cell type quantitated in the filtered snRNA-seq (left) or snATAC-seq dataset (right). PT-proximal tubule, PT_VCAM1-proximal tubule, VCAM1+, PEC-parietal epithelial cells, TAL-thick ascending limb, DCT1-distal convoluted tubule segment 1, DCT2-distal convoluted tubule segment 2, CNT-connecting tubule, PC-principal cells, ICA-intercalated cells type A, ICB-intercalated cells type B, PODO-podocytes, ENDO-endothelial cells, MES-mesangial cells, FIB-fibroblasts, LEUK-leukocytes. The cell type proportions across individuals are also shown as dots. Box-and-whisker plots depict the median, quartiles and range.

Supplementary Table 1 – Patient Demographics, Laboratory Data, and Renal Pathology								
ID	Age	Race	Sex	eGFR (ml/min/1.73m^2)	sCr (mg/dL)	Glomerulosclerosis	IFTA	ANS
Healthy 1	54	NHW	M	58	1.28	None, < 10%	1-10%	Mild
Healthy 2	62	HIS	M	61	1.21	None, < 10%	1-10%	Moderate
Healthy 3	61	NHW	F	69	0.89	None, < 10%	1-10%	Mild
Healthy 4	50	NHW	M	78	1.10	None, < 10%	1-10%	Moderate
Healthy 5	52	NHW	F	98	0.89	None, < 10%	1-10%	Mild

Supplementary Table 1 – Patient demographics and clinical information abstracted from the medical record: Histologic review was performed by a renal pathologist. NHW-non-hispanic white, HIS-hispanic or latino, IFTA-interstitial fibrosis and tubular atrophy, ANS-arterial and arteriolar nephrosclerosis.

Supplementary Table 2<br>Filtered snRNA-seq Dataset		
**Cell Identity**	**Number**	**Frequency**
PT	5036	25.2%
PT_VCAM1	449	2.2%
PEC	552	2.8%
TAL	4435	22.2%
DCT1	2761	13.8%
DCT2	489	2.4%
CNT	1805	9.0%
PC	1022	5.1%
ICA	1107	5.5%
ICB	349	1.7%
PODO	463	2.3%
ENDO	1008	5.0%
MES	239	1.2%
FIB	207	1.0%
LEUK	63	0.3%
**TOTAL**	**19985**	100%

Supplementary Table 2 – The number or frequency of cells for each cell type quantitated in the filtered snRNA-seq dataset: PT-proximal tubule, PT_VCAM1-proximal tubule, VCAM1+, PEC-parietal epithelial cells, TAL-thick ascending limb, DCT1-distal convoluted tubule segment 1, DCT2-distal convoluted tubule segment 2, CNT-connecting tubule, PC-principal cells, ICA-intercalated cells type A, ICB-intercalated cells type B, PODO-podocytes, ENDO-endothelial cells, MES-mesangial cells, FIB-fibroblasts, LEUK-leukocytes. See also Supplementary Fig.20.

Supplementary Table 3<br>Filtered snATAC-seq Dataset		
**Cell Identity**	**Number**	**Frequency**
PCT	6268	23.2%
PST	4280	15.8%
PT_VCAM1	674	2.5%
PEC	403	1.5%
TAL	7762	28.7%
DCT	2777	10.3%
CNT	898	3.3%
PC	1302	4.8%
ICA	611	2.3%
ICB	620	2.3%
PODO	135	0.5%
ENDO	759	2.8%
MES_FIB	352	1.3%
LEUK	193	0.7%
**TOTAL**	**27034**	100%

Supplementary Table 3 – The number of cells or frequency for each cell type quantitated in the filtered snATAC-seq dataset: PCT-proximal convoluted tubule, PST-proximal straight tubule, PT_VCAM1-proximal tubule VCAM1+, PEC-parietal epithelial cells, TAL-thick ascending limb, DCT-distal convoluted tubule, CNT-connecting tubule, PC-principal cells, ICA-intercalated cells type A, ICB-intercalated cells type B, PODO-podocytes, ENDO-endothelial cells, MES_FIB-mesangial cells and fibroblasts, LEUK-leukocytes. See also Supplementary Fig.20.

Supplementary Table 4: Overlap between Cell-type-specific differentially expressed genes and accessible chromatin regions						
snRNA_vs_snATAC	# DEG	DEG with DAR	Prop. DEG with DAR	# DAR	DAR near DEG	Prop. DAR near DEG
PT_vs_PCT	769	333	0.43	3055	618	0.20
PT_vs_PST	769	263	0.34	2273	439	0.19
PT_VCAM1	425	128	0.30	1315	201	0.15
PEC	627	190	0.30	1221	267	0.22
TAL	408	169	0.41	1704	262	0.15
DCT1_vs_DCT	432	178	0.41	1401	277	0.20
DCT2_vs_DCT	348	142	0.41	1401	230	0.16
CNT	442	123	0.28	1146	185	0.16
PC	523	169	0.32	1416	268	0.19
ICA	651	236	0.36	1427	379	0.27
ICB	648	251	0.39	1754	421	0.24
PODO	927	335	0.36	1712	526	0.31
ENDO	861	421	0.49	2781	699	0.25
MES_vs_MES_FIB	774	167	0.22	1203	231	0.19
FIB_vs_MES_FIB	741	179	0.24	1203	248	0.21
LEUK	846	396	0.47	3642	611	0.17
min	348	123	0.22	1146	185	0.15
max	927	421	0.49	3642	699	0.31
mean	636.94	230.00	0.36	1790.88	366.38	0.20
stdev	185.61	94.92	0.08	752.23	166.80	0.04

Supplementary Table 4 – Overlap between cell-type-specific differentially expressed genes and accessible chromatin regions: Cell-type-specific differentially expressed genes (DEG) were identified for each cell type in the snRNA-seq dataset using the Seurat FindAllMarkers function with a log-fold threshold of 0.25 for genes expressed in at least 20% of cells. Cell-type-specific differentially accessible chromatin regions (DAR) were identified for each cell type in the snATAC-seq dataset using the Signac FindAllMarkers function with a log-fold threshold of 0.25 for peaks present in at least 20% of cells. DAR were annotated with the closest gene in the Ensembl database. The annotated gene list was overlapped with DEG to determine the proportion of DEG with a nearby DAR (Prop. DEG with DAR) and the proportion of DAR with a nearby DEG (Prop. DAR near DEG).

Donor	Total RNA reads	RNA reads per cell	Total ATAC reads	ATAC fragments per cell
Healthy_1	499801345	72383	343687555	13892
Healthy_2	317710661	74844	269154397	12611
Healthy_3	391611498	59344	396525222	17493
Healthy_4	368420549	81944	314024252	10567
Healthy_5	310322473	65915	267097032	10168
mean	377573305.2	70886	318097691.6	12946.2
stdev	76365483	8632	54357209	2960

Supplementary Table 5 – The numbers of total reads and reads per nucleus in snRNA-seq and snATAC-seq data: Total reads and reads percell in snRNA-seq and snATAC-seq data were shown.

Quality Control for snRNA libraries		
**Donor**	**Sequencing Saturation**	**Fraction reads with Valid Barcode**
Healthy_1	77.5	89.6
Healthy_2	83.4	77.9
Healthy_3	83	92.5
Healthy_4	82.6	91.7
Healthy_5	80.5	89.7
mean	81.4	88.28
stdev	2.4	5.9

Supplementary Table 6 – Quality control for snRNA-seq libraries: The library complexity for the snRNA libraries was estimated with sequencing saturation for each donor. The fraction of read with a valid barcode in each donor.

**Quality Control for snATAC libraries**		
**Donor**	**Sequencing Saturation**	**Fraction reads with Valid Barcdode**
Healthy_1	36.2	98.3
Healthy_2	35.1	98.3
Healthy_3	37.1	98.3
Healthy_4	41.1	95.8
Healthy_5	37.3	95.8
mean	37.36	97.3
stdev	2.2	1.3

Supplementary Table 7 – Quality control for snATAC-seq libraries: The sequencing saturation and the fraction of reads with a valid barcode in each donor were shown.
