# Supplementary material for: Single cell transcriptional and chromatin accessibility profiling redefine cellular heterogeneity in the adult human kidney
Source: Nat Commun. 2021 Apr 13;12:2190. doi: 10.1038/s41467-021-22368-w (PMC8044133; doi:10.1038/s41467-021-22368-w)
Supplement: Supplementary file 3 — Description of Additional Supplementary Files [file 41467_2021_22368_MOESM3_ESM.pdf]

Description of Additional Supplementary Files

File Name: Supplementary Data 1
Description: Differentially expressed genes in each cell type

File Name: Supplementary Data 2
Description: Differentially accessible regions in each cell type

File Name: Supplementary Data 3
Description: Differentially activated chromVAR motifs in each cell type

File Name: Supplementary Data 4
Description: Genes that were evaluated for allele-specific expression. The ‘pseudo-bulk’ analysis with all cells aggregated together is designated as “ALLCELLS”. Individual cell types and clusters are designated with the corresponding labels (PT=proximal tubule, DCTPC=distal convoluted tubule and principal cells, ICA=type A intercalated cells, ICB=type B intercalated cells, PODO=podocytes, LOH=loop of Henle). Complementary analyses were performed using SNV that map to coding transcripts “mrna” OR “premrna”, which includes intronic reads. p-values are adjusted with Benjamini Hochberg.

File Name: Supplementary Data 5
Description: Differentially expressed genes in PT_VCAM1 vs PT

File Name: Supplementary Data 6
Description: GO enrichment analysis for differentially expressed genes in PT_VCAM1 vs PT

File Name: Supplementary Data 7
Description: Primers used for ChIP-qPCR
