# Supplementary material for: Single cell transcriptional and chromatin accessibility profiling redefine cellular heterogeneity in the adult human kidney
Source: Nat Commun. 2021 Apr 13;12:2190. doi: 10.1038/s41467-021-22368-w (PMC8044133; doi:10.1038/s41467-021-22368-w)
Supplement: Supplementary file 11 — Reporting Summary [file 41467_2021_22368_MOESM11_ESM.pdf]

nature research

Corresponding author(s): Benjamin D. Humphreys

Last updated by author(s): 02/24/2021

Reporting Summary

Nature Research wishes to improve the reproducibility of the work that we publish. This form provides structure for consistency and transparency in reporting. For further information on Nature Research policies, see our Editorial Policies and the Editorial Policy Checklist.

Statistics

For all statistical analyses, confirm that the following items are present in the figure legend, table legend, main text, or Methods section.

n/a	Confirmed	
☐	☒	The exact sample size (n) for each experimental group/condition, given as a discrete number and unit of measurement
☐	☒	A statement on whether measurements were taken from distinct samples or whether the same sample was measured repeatedly
☐	☒	The statistical test(s) used AND whether they are one- or two-sided<br>*Only common tests should be described solely by name; describe more complex techniques in the Methods section.*
☐	☒	A description of all covariates tested
☐	☒	A description of any assumptions or corrections, such as tests of normality and adjustment for multiple comparisons
☐	☒	A full description of the statistical parameters including central tendency (e.g. means) or other basic estimates (e.g. regression coefficient) AND variation (e.g. standard deviation) or associated estimates of uncertainty (e.g. confidence intervals)
☐	☒	For null hypothesis testing, the test statistic (e.g. F, t, r) with confidence intervals, effect sizes, degrees of freedom and P value noted<br>*Give P values as exact values whenever suitable.*
☒	☐	For Bayesian analysis, information on the choice of priors and Markov chain Monte Carlo settings
☐	☒	For hierarchical and complex designs, identification of the appropriate level for tests and full reporting of outcomes
☒	☐	Estimates of effect sizes (e.g. Cohen's d, Pearson's r), indicating how they were calculated

Our web collection on statistics for biologists contains articles on many of the points above.

Software and code

Policy information about availability of computer code

Data collection	Mapping and generation of feature/peak-barcode matrix from fastq files was performed with the use of CellRanger (v3.1.0)/CellRangerATAC (v1.2.0).
Data analysis	Data analysis as described in Method section was performed in R (3.6.1) using the following software packages: Signac (version 0.2.1); Seurat (version 3.0.2); Monocle (Version 3); Cicero (v1.2); chromVAR (v1.6.0); DoubletFinder (v2.0.2); Harmony (v1.0); ChIPSeeker(v1.24.0); clusterProfiler (v3.16.1); STAR (v.2.7.5); GSEA (v4.0.3), BisqueRNA (v1.0.4); DESeq2 (v1.30.1); tximport (v1.16.1); GenomicRanges (v1.40.0), GATK (v4.1.8.1), ASEP (v0.1.0), WASP (v0.3.4), VariantAnnotation (v3.1.2), BWA (v0.7.15), Samtools (v1.9), bcftools (v1.7). All analysis code is available on GitHub at https://github.com/p4rkerw/Muto_Wilson_NComm_2020 and has been deposited in Zenodo at https://doi.org/10.5281/zenodo.4555693.

For manuscripts utilizing custom algorithms or software that are central to the research but not yet described in published literature, software must be made available to editors and reviewers. We strongly encourage code deposition in a community repository (e.g. GitHub). See the Nature Research guidelines for submitting code & software for further information.

Data

Policy information about availability of data

All manuscripts must include a data availability statement. This statement should provide the following information, where applicable:

- Accession codes, unique identifiers, or web links for publicly available datasets
- A list of figures that have associated raw data
- A description of any restrictions on data availability

All relevant data are available from the corresponding authors on reasonable request. Sequencing data is deposited in GEO under accession number GSE151302. Previously-published snRNA-seq data for three adult kidneys are available in GEO (GSE131882). Data tracks for cis-coaccessibility networks and cell-specific

differentially accessible chromatin are available for download and viewing with the UCSC genome browser at (https://genome.ucsc.edu/s/parkercwilson/control_celltype_cr) (Supplementary Fig. 12). Gene expression, ATAC peaks, gene activities and motif activities for each cell type are also available via our interactive website; Kidney Interactive Transcriptomics (http://humphreyslab.com/SingleCell/) (Supplementary Fig. 18). Source data are provided with this paper. Public data repositories used for our analyses include Ensembl http://useast.ensembl.org/index.html , GeneHancer https://www.genecards.org/ , and JASPAR http://jaspar.genereg.net/. Data tracks for the cis-coaccessibility networks and cell-specific differentially accessible chromatin are available for download and viewing with the UCSC genome browser(https://genome.ucsc.edu/s/parkercwilson/control_celltype_cr).
Gene ontology enrichment was performed with PANTHER (http://geneontology.org/). For the TCGA (The Cancer Genome Atlas) dataset, counts and metadata were downloaded from the GDC data portal (portal.gdc.cancer.gov) by selecting “kidney”, “TCGA”, “RNA-seq”, and “solid tissue normal”.

Field-specific reporting

Please select the one below that is the best fit for your research. If you are not sure, read the appropriate sections before making your selection.

☒ Life sciences ☐ Behavioural & social sciences ☐ Ecological, evolutionary & environmental sciences

For a reference copy of the document with all sections, see nature.com/documents/nr-reporting-summary-flat.pdf

Life sciences study design

All studies must disclose on these points even when the disclosure is negative.

Sample size	No sample size calculation was performed. Samples from 5 individuals were processed to obtain ~6,000 single nuclei per sample. The number of nuclei analyzed in this study was sufficient to draw the conclusions described in the manuscript.
Data exclusions	For snATAC-seq, low-quality cells were removed from the aggregated snATAC-seq library (peak region fragments > 2500, peak region fragments < 25000, %reads in peaks > 15, blacklist ratio < 0.001, nucleosome signal < 4 & mitochondrial gene ratio < 0.25) before normalization with term-frequency inverse-document-frequency (TFIDF). For snRNA-seq, we removed low-quality nuclei (Features > 500, Features < 4000, RNA count < 16000, %Mitochondrial genes < 0.8, %Ribosomal protein large or small subunits < 0.4), and then we used DoubletFinder v2.0.2 (28) to remove heterotypic doublets (assuming 6% of barcodes represent doublets).
Replication	For snRNA-seq or snATAC-seq; samples from 5 individuals were processed independently to generate libraries and similar results were obtained. Immunohistochemistry (Fig. 4c and 5c) or immunofluorescence studies (Fig.5a, b, d and Fig. 6e) were performed on n = 3 samples at least two times, and similar results were obtained. The quantifications in immunofluorescence data were performed in five 200x images randomly taken from each patient (n = 3). Three independent experiments were performed for ChIP-qPCR experiments, and similar results were obtained (Fig. 6f and Fig. S8).
Randomization	Randomization is not relevant to this study because there was a single experimental group with 5 samples.
Blinding	No blinding was used during data collection or analysis. It was not considered necessary for the study, because the measurements could not be affected by the operator.

Reporting for specific materials, systems and methods

We require information from authors about some types of materials, experimental systems and methods used in many studies. Here, indicate whether each material, system or method listed is relevant to your study. If you are not sure if a list item applies to your research, read the appropriate section before selecting a response.

Materials & experimental systems

n/a	Involved in the study	
☐	☒	Antibodies
☐	☒	Eukaryotic cell lines
☒	☐	Palaeontology and archaeology
☒	☐	Animals and other organisms
☐	☒	Human research participants
☒	☐	Clinical data
☒	☐	Dual use research of concern

Methods

n/a	Involved in the study	
☒	☐	ChIP-seq
☒	☐	Flow cytometry
☒	☐	MRI-based neuroimaging

Antibodies

Antibodies used	The following primary antibodies were used for immunofluorescence studies.<br>Antigen; Clone; Manufacturer; Catalogue number; Final dilution<br>VCAM1 (EPR5047; abcam; ab134047; 1/200)<br>biotinylated Lotus Tetragonolobus Lectin (NA; Vector Laboratories; B-1325; 1/200)<br>HNF4A (H-1; Santa Cruz Biotechnology; sc-374229; 1/200)<br>CD24 (SN-3; Santa Cruz Biotechnology; sc-19585; 1/20)

CD133 (AC133; Miltenyi Biotec; 130-090-422; 1/10).
UMOD (Bio-Rad; 8595-0054; 1/200)
These sections were subsequently stained with secondary antibodies listed below
Alexa488-conjugated donkey anti-rabbit antibody (Jackson ImmunoResearch; 711-545-152; 1/200).
Cy3-conjugated donkey anti-sheep antibody (Jackson ImmunoResearch; 713-165-147; 1/200).
Cy3-conjugated goat anti-mouse antibody (Jackson ImmunoResearch; 115-165-003; 1/200).
Alexa647-conjugated strepavidin (Jackson ImmunoResearch; 016-600-084; 1/200).

The following primary antibodies were used for immunohistochemical studies.
Antigen; Clone; Manufacturer; Catalogue number; Final dilution
SLC12A1 (Sigma-Aldrich; HPA014967; 1/100)
VCAM1 (abcam; EPR5047; ab134047; 1/200)
Aquaporin 1 (Abcam; Ab15080; 1/100)
SLC34A1 antibody (Not commercially available; Clone 16; undiluted culture supernatant)
PTH1R (R&D systems; AF5709; 1/100)
UMOD (Biotrend; BT85-9500-54; 1/1000)
KCNJ10 (Alamone; APC-035; 1/1000)
These sections were subsequently stained with the secondary antibodies listed below
Rabbit Anti-Goat Immunoglobulins HRP conjugated (Dako, P0449; 1/200)
Goat Anti-Mouse Immunoglobulins HRP conjugated (Dako, P0447; 1/200)
Goat Anti-Rabbit Immunoglobulins HRP conjugated (Dako, P0448; 1/200)

The following primary antibodies were used for ChIP-qPCR.
Antigen; Clone; Manufacturer; Catalogue number; Final dilution
RELA (Sigma-Aldrich; 17-10060; 1/250)
HNF4A (EPR16885; Abcam; ab181604; 1/250)

Validation

The antibodies except SLC34A1 antibody (Clone 16) were commercially available with validations described on the associated company websites. SLC34A1 antibody (Clone 16) was validate in the previous literature referenced in the MS (PMID: 31241991). The other antibodies were validated by the manufacturer as below.
Antigen; Species; Application; Link

VCAM1 (mouse, rat, human; WB, IP, IHC-P, Flow Cyt, ICC/IF, ELISA; https://www.abcam.com/vcam1-antibody-epr5047-ab134047.html)
HNF4A (mouse, rat and human; Western Blottin, immunoprecipitation, immunofluorescence, immunohistochemistry (including paraffin-embedded sections), ELISA; https://datasheets.scbt.com/sc-374229.pdf)
CD24 (mouse, rat and human; Western Blotting, immunoprecipitation, immunofluorescence and flow cytometry; https://datasheets.scbt.com/sc-19585.pdf)
CD133 (human; FC, MC, ICC, WB; https://www.miltenyibiotec.com/US-en/products/cd133-1-antibody-anti-human-ac133.html#gref; In order to compare the epitope specificity of an antibody, the clone being used is compared with other known clones recognizing the same antigen in a competition assay)
UMOD (human and Mouse; ELISA, Immunofluorescence, Immunohistology - Frozen; https://www.bio-rad-antibodies.com/polyclonal/human-tamm-horsfall-glycoprotein-antibody-8595-0054.html?f=purified)
biotinylated Lotus Tetragonolobus Lectin (NA; Immunohistochemistry / Immunocytochemistry, Immunofluorescence, Blotting Applications, Elispot, ELISAs, Glycobiology; https://vectorlabs.com/biotinylated-lotus-tetragonolobus-lectin-ltl.html)
Alexa488-conjugated donkey anti-rabbit antibody (Rabbit; https://www.jacksonimmuno.com/catalog/products/711-545-152).
Cy3-conjugated donkey anti-sheep antibody (Sheep; https://www.jacksonimmuno.com/catalog/products/713-165-147).
Cy3-conjugated goat anti-mouse antibody (Goat; https://www.jacksonimmuno.com/catalog/products/115-165-003).
Alexa647-conjugated strepavidin (Biotin; https://www.jacksonimmuno.com/catalog/products/016-600-084).
SLC12A1 (human; immunohistochemistry; https://www.sigmaaldrich.com/catalog/product/sigma/hpa014967?lang=en&region=US; developed and validated by the Human Protein Atlas (HPA) project)
Aquaporin 1 (Human; ELISA; https://www.abcam.com/aquaporin-1-antibody-ab15080.html)
PTH1R (Human; immunohistochemistry, western blot; https://www.rndsystems.com/products/human-pth1r-pthr1-antibody_af5709)
UMOD (Human; https://www.biotrend.com/-186/sheep-polyclonal-anti-human-tamm-1206501053.html)
KCNJ10 (H, M, R; ICC, IF, IHC, IP, WB; https://www.alomone.com/p/anti-kir4-1/APC-035)
Rabbit Anti-Goat Immunoglobulins HRP conjugated (https://www.agilent.com/store/en_US/Prod-P044901-2/P044901-2)
Goat Anti-Mouse Immunoglobulins HRP conjugated (https://www.agilent.com/store/productDetail.jsp?catalogId=P044701-2)
Goat Anti-Rabbit Immunoglobulins HRP conjugated (https://www.agilent.com/store/productDetail.jsp?catalogId=P044801-2)
RELA (H, M, R; WB, ChIP; https://www.emdmillipore.com/US/en/product/ChIPAb+-NFB-p65-RelA-ChIP-Validated-Antibody-and-Primer-Set,MM_NF-17-10060)
HNF4A (Mouse, Rat, Human; ChIP, IHC-P, WB, IP; https://www.abcam.com/hnf-4-alpha-antibody-epr16885-chip-grade-ab181604.html)

Eukaryotic cell lines

Policy information about cell lines

Cell line source(s)

Primary RPTEC was purchased from Lonza (https://bioscience.lonza.com/lonza_bs/US/en/Primary-and-Stem-Cells/p/000000000000185055/RPTEC-%E2%80%93-Human-Renal-Proximal-Tubule-Epithelial-Cells).

Authentication

Not authenticated by the authors.

Mycoplasma contamination

All cells were negative for Mycoplasma contamination.

Commonly misidentified lines (See ICLAC register)	No commonly misidentified cell lines were used.

Human research participants

Policy information about studies involving human research participants

Population characteristics	Supplemental Table 1 lists the metadata for the patients from which the data were generated. There was no genotype information available from the participants.
Recruitment	5 human kidney tissues from nephrectomy were used with informed consent.
Ethics oversight	Sample collection for sequencing was performed in Brigham and Women's Hospital (Boston, MA) under an established Institutional Review Board protocol. For sample collection for immunohistochemistry, Informed written consent was obtained from each patient and approved by the Biomedical Research Ethics Committee of Southern Denmark in accordance with the Declaration of Helsinki.

Note that full information on the approval of the study protocol must also be provided in the manuscript.
